# Early and Middle Holocene Hunter-Gatherer Occupations in Western Amazonia: The Hidden Shell Middens

**DOI:** 10.1371/journal.pone.0072746

**Published:** 2013-08-28

**Authors:** Umberto Lombardo, Katherine Szabo, José M. Capriles, Jan-Hendrik May, Wulf Amelung, Rainer Hutterer, Eva Lehndorff, Anna Plotzki, Heinz Veit

**Affiliations:** 1 Institute of Geography, University of Bern, Bern, Switzerland; 2 School of Earth & Environmental Sciences, University of Wollongong, Wollongong, Australia; 3 Center for Comparative Archaeology, University of Pittsburgh, Pittsburgh, Pennsylvania, United States of America; 4 Institute of Crop Science and Resource Conservation, University of Bonn, Bonn, Germany; 5 Zoologisches Forschungsmuseum Alexander Koenig, Bonn, Germany; New York State Museum, United States of America

## Abstract

We report on previously unknown early archaeological sites in the Bolivian lowlands, demonstrating for the first time early and middle Holocene human presence in western Amazonia. Multidisciplinary research in forest islands situated in seasonally-inundated savannahs has revealed stratified shell middens produced by human foragers as early as 10,000 years ago, making them the oldest archaeological sites in the region. The absence of stone resources and partial burial by recent alluvial sediments has meant that these kinds of deposits have, until now, remained unidentified. We conducted core sampling, archaeological excavations and an interdisciplinary study of the stratigraphy and recovered materials from three shell midden mounds. Based on multiple lines of evidence, including radiocarbon dating, sedimentary proxies (elements, steroids and black carbon), micromorphology and faunal analysis, we demonstrate the anthropogenic origin and antiquity of these sites. In a tropical and geomorphologically active landscape often considered challenging both for early human occupation and for the preservation of hunter-gatherer sites, the newly discovered shell middens provide evidence for early to middle Holocene occupation and illustrate the potential for identifying and interpreting early open-air archaeological sites in western Amazonia. The existence of early hunter-gatherer sites in the Bolivian lowlands sheds new light on the region’s past and offers a new context within which the late Holocene “Earthmovers” of the Llanos de Moxos could have emerged.

## Introduction

The peopling of Amazonia and the adaptive strategies of its early inhabitants are among the least known aspects of the archaeology of the New World. Late Pleistocene and early Holocene sites have been documented in several environmentally-diverse locations in South America [1]. Most of these early sites have been found along the continental coastland [2]–[6], where the predictability of resources facilitated the initial dispersion of humans [7]. Hunter-gatherer sites associated with extinct fauna have been found in the cool, sub-humid conditions of southern South America [8], [9]. Ancient South Americans flourished in the “cerrado” and “caatinga” environments of the Brazilian highlands, where a diversity of animals was available for hunting [10]. Evidence for human occupation of the Andes is also abundant [11]–[13]. Here, vertical gradients in climate, vegetation and hydrology create a wide variety of environments accessible within short distances [14]. However, very few early sites have been found in Amazonia, the most important being: Peña Roja, an open-air site located on a riverine terrace in south eastern Colombia [15] and Caverna de Pedra Pintada, a rock shelter located downstream from the confluence of the Tapajós and Amazon rivers [16]. These sites are found in a rainforest environment and are characterized by the presence of flakes and other stone tools [17]. The absence of other early human settlements in most of the Amazon Basin has been attributed to unfavourable environmental conditions [18], [19] and sampling bias [1], [16]. Archaeological research in Amazonia has mostly focused on horticultural groups [20]. Recent studies have documented evidence of late Holocene Amazonian complex societies in southwestern Peru, central Brazil and the Llanos de Moxos in Bolivia, shedding new light on the ongoing debate about the nature and scale of pre-Columbian occupation [21]–[24]. Even though this work has led to increasing consensus about the level of social complexity that developed in this region, the origins of these societies remain to a large extent archaeologically unexplored. Moreover, the antiquity, extent and nature of human presence in this region before the late Holocene are still unknown.

**Figure 1:**
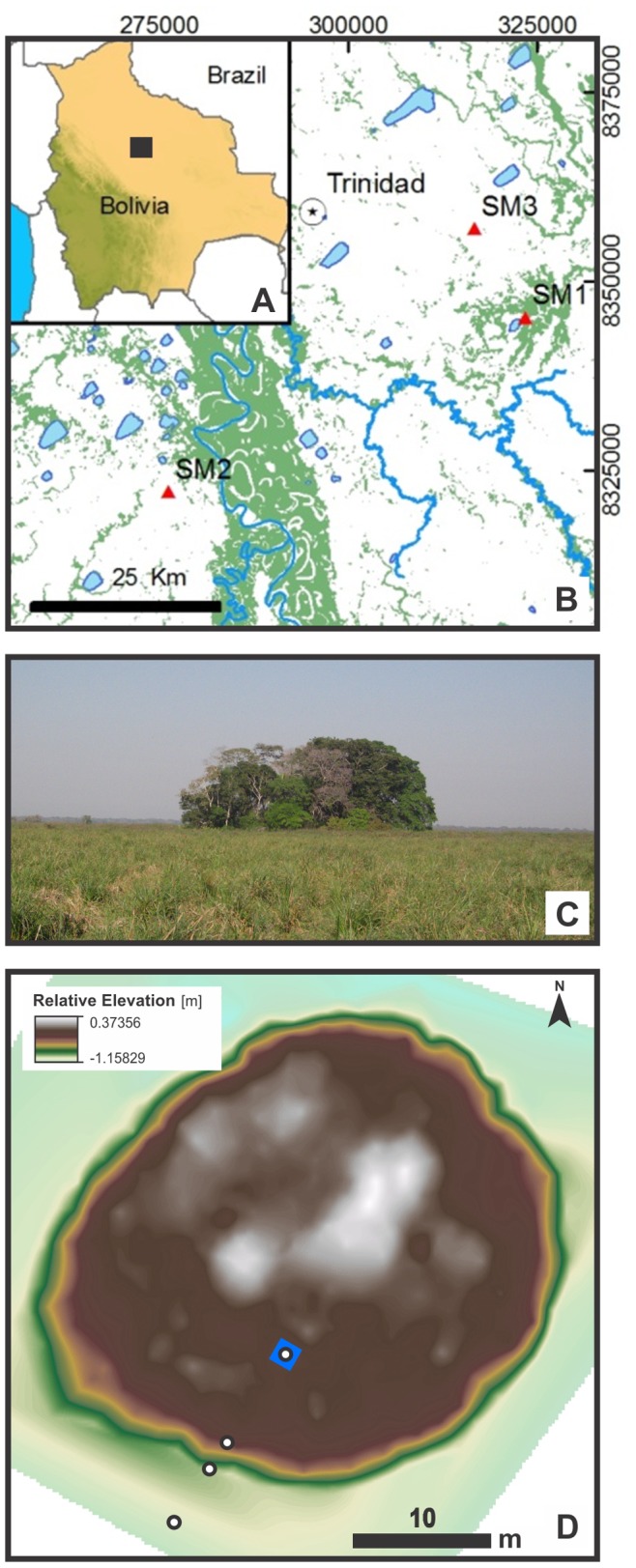
Geographic location of the three forest islands investigated (SM1, SM2 and SM3). a) Bolivian lowlands; b) the Llanos de Moxos; c) Panorama of SM1 (Isla del Tesoro) and surrounding savannah (view towards the SW); d) Digital Elevation Model of SM1 with the location of the 1×1 m test excavation (blue box) and some of the cores (white points) shown in Fig. 3.

Our study focuses on the Llanos de Moxos (LM), in the Bolivian Amazon (Fig. 1a). The region holds an impressive number of late Holocene pre-Columbian earthworks, including hundreds of large earthen mounds and thousands of kilometres of raised fields and sophisticated drainage works, suggesting that the region was able to support relatively large populations in the past [25], [26]. The LM is among the largest inundated tropical savannah landscapes in the world [27], [28]. Today, these plains are characterized by a forest–savannah mosaic resulting from seasonal inundations [29]. River discharge, fluvial sedimentation processes, and inundation patterns are highly variable in space and time [27], [30]–[32]. The complex network of active and inactive river channels suggests that in the past the fluvial system was highly dynamic [33].

Hundreds of forest islands – small forested earthen mounds of diverse origin – are found scattered throughout the LM [34], [35]. Little is known about the origin of these forest islands; their existence has been attributed to natural processes such as termite mound building [36], [37] and erosional fragmentation of older surfaces [28], [34], [38], alternatively, some authors have attributed their origin to the anthropogenic activities of the late Holocene pre-Columbian “Earthmovers” societies [26], [34], [39]. Whilst it is likely that some forest islands are of natural origin and some were built by late-Holocene peoples, this paper reports on the discovery of three forest islands that are, in fact, early and middle Holocene anthropogenic shell middens (SM1, SM2 and SM3 in Fig.1) and presents detailed geoarchaeological analysis of one of these sites: SM1, locally known as Isla del Tesoro. Archaeological remains at SM1 and SM3 date back to the early Holocene, making them the oldest sites in western Amazonia and two of the oldest shell middens in South America. Some of the best known shell midden mounds are located in southeastern coastal Brazil, where they are known as *sambaquis*. However, the oldest *sambaquis*, which date to the early Holocene (ca. 10000 cal BP), are riverine; this has led researchers to suggest that earlier coastal *sambaquis* were probably inundated by the sea level rise that followed the transition to the Holocene [6]. Coastal populations could have begun to expand along rivers at this time [6]. An early Holocene shell mound has also been reported in the lower Amazonas [40], suggesting the existence of this kind of settlements in other parts of tropical South America.

Here, we present the first results of a geoarchaeological analysis of early and middle Holocene shell middens in the LM, thousands of kilometres from the locations of previously known shell middens. We discuss the implications of this discovery for understanding the diversity of occupation witnessed in early South America as well as the later pre-Columbian archaeological record of the LM.

## Materials and Methods

**Figure 2:**
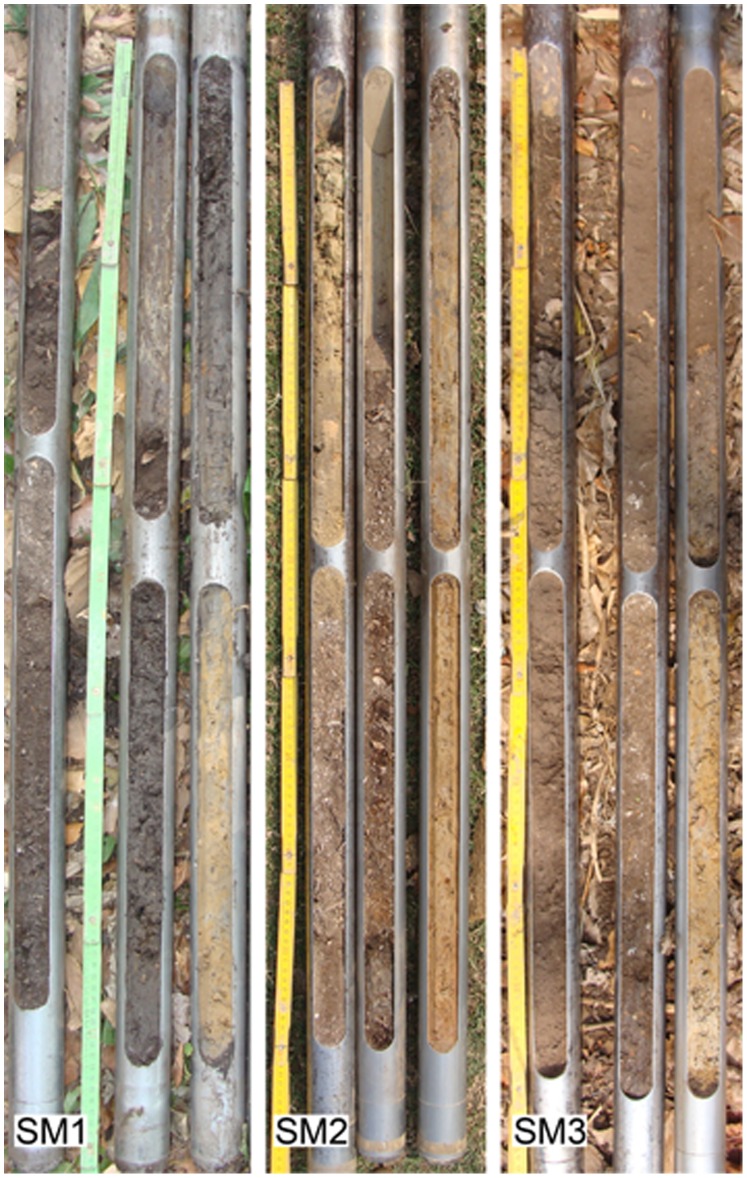
Photographs of the 3 meter cores extracted from the SM1, SM2 and SM3 sites. The thickness of the anthropogenic sediments is 2.5, 1.7 and 2.5 meters respectively. In SM2 the first 50 cm are made of sediments that have been deposited on top of the archaeological site in modern times.

The study looks at three sites (SM1, SM2 and SM3 in Fig. 1b). For SM2 and SM3 only two minimally invasive cores were extracted with a Wacker vibracorer in order to recover material for radiocarbon dating and gain an overall idea of their stratigraphy (Fig. 2). In the case of SM1 (Isla del Tesoro) an exploratory 1×1 meter pit was excavated to a depth of 1.7 meters, where the appearance of the water table halted further excavation. About 500 gr. of material was sampled at ten centimetre intervals and stored in plastic bags. The stratigraphy and the relationship between SM1, a buried paleosol abutting it and the surrounding sediments have been established based on a transect of sediment cores extracted across the midden-savannah boundary. The chronology of the formation and abandonment of SM1 is based on 28 radiocarbon ages (Table 1). Radiocarbon ages have been measured at Poznan Radiocarbon Laboratory and were calibrated using Calib 6.0 [41] and the ShCal04 calibration curve [42]. Despite the variable condition of the shell fragments, paired dates from shell fragments and charcoal throughout the sequence are in close agreement, and show that problems with unpredictable reservoir effects in the radiocarbon dating of freshwater shell [43], [44] do not significantly affect our chronology. 30 µm ( = 0.03 mm) thin sections with cover slips have been prepared following standard procedures by Geoprep at the Department of Earth Sciences, University of Basel. Samples have been impregnated with epoxy and then cut vertically in order to maintain consistency with the original stratigraphic position. Soil organic carbon (C_org_) has been measured with a C/N analyser after removal of carbonates with HCl 10%. The total amount of carbonates has been measured by loss on ignition as W950°–W550° after 2 hours at 550°C and 2 hours at 950°C. The respective contribution of aragonite and calcite to the total amount of carbonates has been measured by powder x-ray diffraction (XRD) using a Philips PW 1800. Steroid composition has been analysed by gas-chromatography and mass spectrometry (GC-MS) (Figure S1, Table S1 and S2) after accelerated solvent extraction with DCM:MeOH 9∶1 (v/v), derivatization with BSTFA and pyridine (see Text S1 for further details on the methods). The content of black carbon (BC) has been estimated after converting condensed aromatic moieties to benzene-polycarboxylic acids as specific BC markers [45], [46] (Table 2; see Text S1 for further details on the methods). Multi-element analysis has been performed by x-ray fluorescence (XRF) (Table 3). For each sample, 4 g of dried and milled (vibration disk mill) sample material was mixed with 0.9 g of wax (Licowax C Micropowder PM) and then pressed to a pellet at 10–15 tons. Elemental composition was determined with the Phillips 2400 x-ray fluorescence spectrometer. The semi-quantitative measurements were carried out with the UniQuant program. Producing quantitative datasets for the faunal remains proved difficult due to the uneven nature of preservation. Cemented blocks of fragmented shell ruled out any form of quantification involving counting and also distorted weights. Likewise, carbonate concretions on many of the bones rendered quantification by weight spurious. In acknowledgement of these limitations, Table S3 presents a partially quantitative and partially qualitative description of the faunal assemblage.

**Table 1:** List of radiocarbon ages for sites SM1, SM2, SM3 and paleosol.

Sample name	Lab. No.	Site	Depth below ground [cm]	Dated material	^14^C age	Cal. yr BP 95.4%	RAUPD
IT 15	Poz-34228	SM1	15	Bone	345±25	401±51	0.780
IT 30/40	Poz-34229	SM1	35	Shell	3895±35	4280±132	0.981
IT 40/50	Poz-34230	SM1	45	Shell	4945±35	5652±68	0.986
IT 08 45–50	Poz-28854	SM1	48	Shell	3830±50	4140±161	0.965
IT 08 77	Poz-28855	SM1	77	Charcoal	4415±35	4918±70	0.867
IT 115 (char)	Poz-22902	SM1	115	Charcoal	5520±40	6253±71	0.949
IT 115	Poz-24633	SM1	115	Shell	5360±40	6097±112	0.940
IT 110/120	Poz-34231	SM1	115	Shell	5520±40	6253±71	0.949
IT 120	Poz-24634	SM1	120	Charcoal	5505±35	6248±66	0.990
IT 120/130	Poz-34232	SM1	125	Shell	5460±40	6235±62	0.780
IT 08 SF2 140	Poz-28856	SM1	140	Charcoal	4480±40	4973±106	0.704
IT 08 140	Poz-28850	SM1	140	Shell	4495±35	4979±105	0.601
EXC 160	Poz-36136	SM1	165	Charcoal	5800±35	6549±105	1
ITD 230/240	Poz-34301	SM1	235	Bulk organics	9270±60	10382±141	0.975
ITD –245	Poz-36135	SM1	245	Charcoal	9420±50	10604±126	0.938
166/80–90	Poz-38853	SM2	85	Shell	4950±40	5657±73	0.981
166/150–160	Poz-38850	SM2	155	Charcoal	4770±60	5508±77	0.566
166/205 (1)	Poz-38851	SM2	205	Shell	5380±40	6103±112	0.942
166/205 (2)	Poz-38852	SM2	205	Charcoal	5500±40	6248±71	0.957
191/105–110	Poz-38862	SM3	107	Shell	5140±40	5824±100	1
191/233–239	Poz-38865	SM3	236	Shell	7860±50	8578±150	0.989
191/238–243	Poz-38866	SM3	240	Charcoal	7790±80	8549±172	1
GPS52 154/161	Poz-34303	Paleosol	155	Bulk organics	6200±40	7038±128	1
170/224–226	Poz-38855	Paleolos	225	Bulk organics	5610±50	6363±86	0.989
Beni 35 NaOH	Poz-22766	Paleosol	100	Humic acids	4305±35	4832±43	0.613
Beni 35 RES	Poz-22767	Paleosol	100	Humin	4545±35	5167±134	0.936
205/292–295	Poz-38867	Paleosol	293	Bulk organics	5070±40	5776±123	1
205/369–371	Poz-38868	Paleosol	370	Bulk organics	5840±50	6572±123	0.974

RAUPD stands for Relative Area Under Probability Distribution, values are based on the SHCal04 Southern Hemisphere calibration curve [42].

* For the location of the paleosol samples see Figure S2.

**Table 2:** Amount of black carbon (BC) in SM1 and in the surrounding paleosol.

Unit	Lat	Long	Sample	Depth below ground [cm]	BC-C [g C kg^−1^]	BC-C [g C kg^−1^ C_org_]
I			15526	5	7.0	106.2
I			15527	15	9.0	218.2
I			15528	25	7.0	210.0
III			15529	35	4.1	129.3
III			15530	45	2.8	163.3
III			15532	75	3.7	215.0
III			15535	85	4.7	273.4
III			15535	85	4.3	247.8
III			15538	115	5.1	283.7
VII			15543	165	6.3	320.7
Paleosol 52	−14.9621	−64.6400	15508	160	1.8	419.8
Paleosol 68	−14.9620	−64.6410	20469	150	2.9	317.5
Paleosol 81	−14.9616	−64.6607	20471	165	1.1	349.7

See Text S1 for more details on methods.

**Table 3:** XRF results for 3 paleosol samples (52,68 and 81) taken from the surroundings of SM1 and from the SM1 (0–170 cm) profile.

Sample	Na2O	MgO	Al2O3	SiO2	Px	SO3	Cl	K2O	CaO	Sc	Ti	V	Cr	Mn	Fe2O3	Cu	Zn	Rb	Sr	Zr	La	Pb
**52**	4.09	11.7	208.7	638.6	0.252	0.534	0.041	24.2	4.68	0	6.12	0.146	0.06	0	25.8	0.019	0.041	0.152	0.107	0.406	0	0.044
**68**	4.37	12.8	210.2	632.6	0.305	0.595	0.026	28	5.69	0	5.4	0.133	0.058	0.094	18.2	0.025	0.039	0.169	0.142	0.39	0	0.044
**81**	3.32	11.7	196.5	625.3	0.274	0.259	0.025	25.4	5.58	0	4.84	0.137	0.072	0.538	25	0.034	0.057	0.176	0.115	0.33	0	0.045
**0–10 U. I**	2.26	10.5	109.9	555.3	12.7	2.28	0.122	13.7	51.2	0	3.2	0.063	0.028	1.2	28	0.05	0.296	0.11	0.198	0.244	0	0.03
**10–20 U. I**	2.7	10.7	119.3	580.2	15	1.4	0.102	14.3	52	0	3.56	0.071	0.028	1.27	30.2	0.043	0.317	0.11	0.209	0.267	0	0.036
**20–30 U. I**	2.47	10.6	121.8	499.9	16.3	1.45	0.069	13.7	114.2	0	3.61	0.095	0.042	1.01	30	0.035	0.261	0.096	0.363	0.238	0	0.035
**30–40 U. IIIa**	2.18	9.68	85.8	368.1	15.4	3.31	0.134	10.4	333.2	0.116	3.09	0.082	0.029	0.884	26.3	0.027	0.259	0.081	0.787	0.204	0.231	0.032
**40–50 U. IIIa**	1.99	10.4	66.9	259.9	13	2.5	0.099	8.51	325.7	0.103	2.41	0.068	0	0.637	17.4	0.03	0.187	0.052	0.74	0.165	0.211	0.025
**50–60 U. IIIa**	2.12	9.05	64.3	259.9	12	2.45	0.106	8.36	327.5	0.129	2.58	0.094	0.038	0.593	17.1	0.029	0.189	0.048	0.761	0.172	0.179	0.022
**60–70 U. IIIa**	1.83	10.2	55.8	226	13.2	2.92	0.092	7.66	356.5	0.117	2.23	0.075	0	0.533	14.6	0.035	0.242	0.044	0.789	0.164	0.245	0.02
**70–80 U. IIIb**	2.09	12.7	69.2	267	19	2.8	0.117	9.43	317.2	0.121	2.62	0.074	0.037	0.941	17.7	0.043	0.296	0.056	0.662	0.185	0.198	0.029
**80–90 U. IIIb**	2.83	12.7	78	300.6	17.6	2.11	0.201	11.1	288.6	0.076	3.01	0.091	0.02	0.847	20.2	0.054	0.295	0.068	0.623	0.202	0.143	0.029
**90–100 U. IIIb**	2.34	11.2	73.1	288.3	14.8	1.69	0.121	11	300.8	0.102	3.06	0.092	0.025	1.2	19.4	0.057	0.264	0.062	0.755	0.224	0.191	0.022
**100–110 U. IIIb**	2.42	9.27	70	272.3	13.6	1.7	0.169	10.6	318	0.102	2.79	0.088	0.028	0.992	18.3	0.049	0.24	0.058	0.895	0.203	0.165	0.026
**110–120 U. IIIb**	2.39	8.49	72.8	289.5	12.2	1.59	0.112	10.7	293	0.095	2.93	0.077	0.036	1.43	18.6	0.036	0.241	0.054	0.912	0.185	0.194	0
**120–130 U. V**	1.29	6.32	9.89	38	11.7	1.19	0.107	1.86	512.5	0.19	0.472	0.078	0	0.991	3.6	0.021	0.388	0	0.82	0.022	0.335	0
**130–140 U. IV**	2.12	3.97	33.1	138.9	13.6	0.93	0.225	5.39	408.2	0.145	1.53	0.086	0.021	0.909	10.1	0.053	0.426	0.027	1.26	0.073	0.256	0
**140–150 U. VI**	1.99	4.54	43	167.1	14.8	0.816	0.102	6.74	393.9	0.114	1.81	0.07	0.029	0.902	11.5	0.04	0.405	0.037	1.11	0.104	0.229	0
**150–160 U. VII**	2.93	7.36	79.6	306.3	25.6	1.01	0.116	12.2	260.3	0.1	2.94	0.081	0.022	0.766	18.9	0.07	0.505	0.06	0.853	0.173	0.194	0.019
**160–170 U. VII**	3.24	8.99	103	392.3	20.3	0.943	0.12	17	201.4	0.069	3.97	0.129	0.036	0.589	25.7	0.06	0.359	0.083	0.702	0.272	0.156	0.032

Values are in g kg^−1^.

The present work has been performed under authorization N° 017/2012 issued by the Unidad de Arqueología y Museos (UDAM) del Estado Plurinacional de Bolivia. All necessary permits were obtained for the described study, which complied with all relevant regulations. We thank the Beni’s Ganaderos for granting us full access to their properties.

## Results

**Figure 3:**
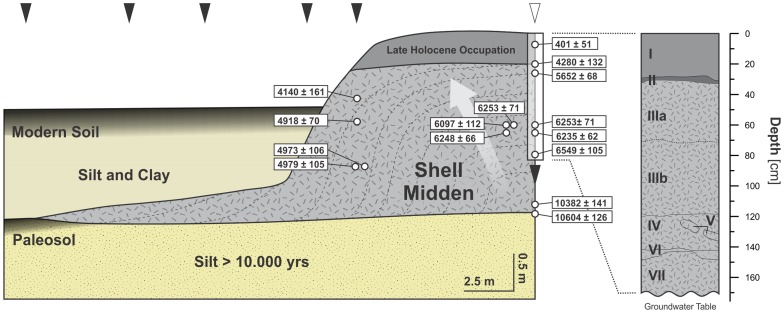
Cross-section transect of the shell midden SM1. Dashed lines and grey arrow highlight the onion-like growth of the midden reflected in the 14C dates. The black triangles above mark the coring locations and the white triangle the excavation site. The location of the transect and excavation is shown in Fig 1d.

**Figure 4:**
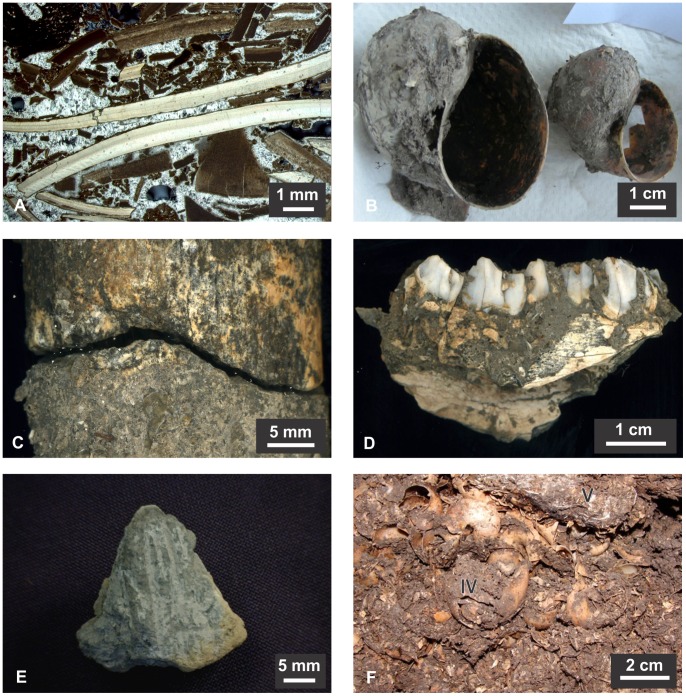
Details of recovered burnt earth, shells and bone remains from excavations at SM1. A) Thin section from Unit VI; aragonitic and micritic shell fragments cemented together; a bone is visible in the upper left corner (cross polarized light, XPL); b) *Pomacea* shells found at a depth of 110 cm; c) Impact scar between refitted fragments of a *Blastocerus dichotomus* tibia found at 160 cm. Mineral dendrites covering the edges of bones and surface damage indicate a percussive blow; d) Mandibular fragment of *Mazama* sp. found at a depth of 70–75 cm; e) Fragment of burnt earth found at a depth of 140 cm with incised parallel lines, probably culturally-modified; f) Units IV and V as observed in the excavations; Unit V is a layer of well cemented shells surrounded by loose fragments that form Unit IV.

**Figure 5:**
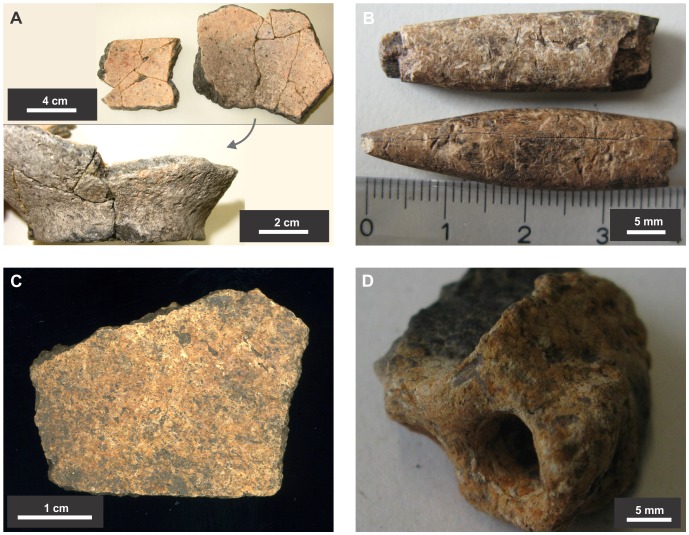
Material retrieved from the uppermost 30 cm of SM1. Unit I, corresponding to the late-Holocene occupation. A) Fragmented pottery; b) Bone tools; c) Fragment of human skull; d) Biogenic burnt earth, probably a wasp chamber.

**Figure 6:**
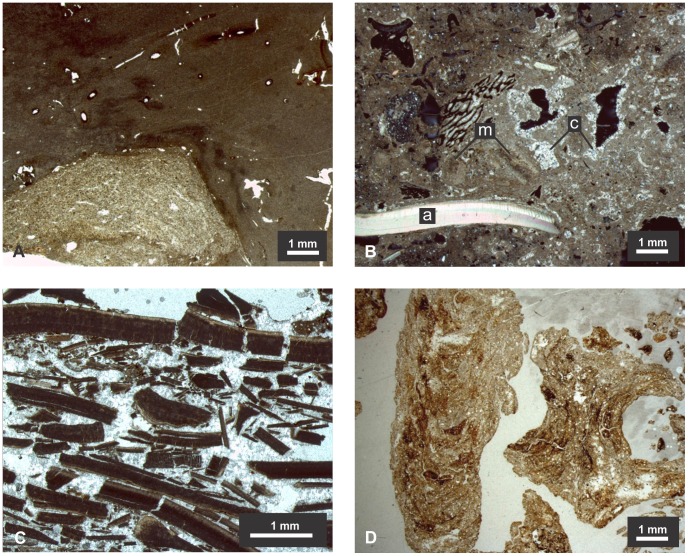
Micromorphology of SM1’s key features and units. A) Fragment of burnt earth from Unit II showing a relatively undisturbed mass of clay with a cluster of coarser material; the association of two different materials suggests reworking, but the presence of root channels indicates it was limited (PPL); b) Unit IIIa at depth 45 cm; “a” indicates an aragonitic shell fragment, “m” indicates recrystallized shell fragments and “c” indicates precipitated calcite crystals (XPL); c) Unit V at depth 130 cm entirely constituted by burnt shell fragments and precipitated calcite (plane polarized light, PPL); d) Biogenic burnt earth fragments at depth of 75 cm; on the left a longitudinal cut of the chamber; on the right a transversal cut of the chamber (PPL).

Cores from the three forest islands selected for sampling, two east of Trinidad (SM1, SM3) and one west of the Mamoré River (SM2) (see Fig. 1b), revealed organic-rich, stratified sequences of accumulated deposits of freshwater gastropod shells belonging to the genus *Pomacea* (Fig. 2). Test excavations at SM1 (Fig. 1c, d) exposed a 1.7 m stratigraphic profile of a midden containing dense accumulations of shells, as well as animal bones and charcoal throughout. The excavation could not go deeper because at 1.7 m we reached the water table. However, a core taken from the bottom of the excavation indicates that the shell midden extended about 80 cm deeper. The base of the midden is found at a depth of about 150 cm below to the current surface of the savannah, where the earliest archaeological materials date to 10604±126 cal BP. The radiocarbon dates generally follow a vertical trajectory, with deeper deposits being older. Assuming an onion-like accretion growth model for SM1 (Fig. 3), the ages between 5000 and 4000 cal BP could indicate a period in which the midden grew in extension rather than in elevation, with each additional mound layer enveloping the last. The radiocarbon chronology and stratigraphic sequence of SM1 (Fig. 3, Table 1) shows two distinct depositional phases: 1) a stratified shell mound (mostly composed of freshwater apple snails, *Pomacea* spp.) (Fig. 4), which grew upward and outward throughout the early and middle Holocene (Units III–VII), and 2) an overlying layer composed of organic refuse containing pottery, bone tools and human bones (Fig. 5), dated to the late Holocene (Unit I). These are separated by a thin layer rich in pieces of burnt clay and earth with diameters of between 2 cm and 6 cm (Fig. 6a) (Unit II). The base of the shell midden rests on an older surface on which a paleosol has formed abutting the midden but not below it. Radiocarbon ages of the paleosol throughout the region (Table 1, Figure S2) indicate that it formed synchronously with the deposition of the midden. The paleosol and the flanks of the shell mound are covered by 1.5 meters of clayey and silty sediments, resulting from a middle Holocene fluvial deposition of the Grande River [22].The cores from the shell middens SM2 and SM3 show an analogous stratigraphic and chronological structure (Fig. 2).

The uppermost 30 cm of the deposits (Unit I in Fig. 3) contained occasional fragments of earthenware pottery. Radiocarbon dating of a Caiman *(Caiman yacare)* cranium fragment with signs of butchery dates this uppermost cultural Unit to 401±51 cal BP.

**Figure 7:**
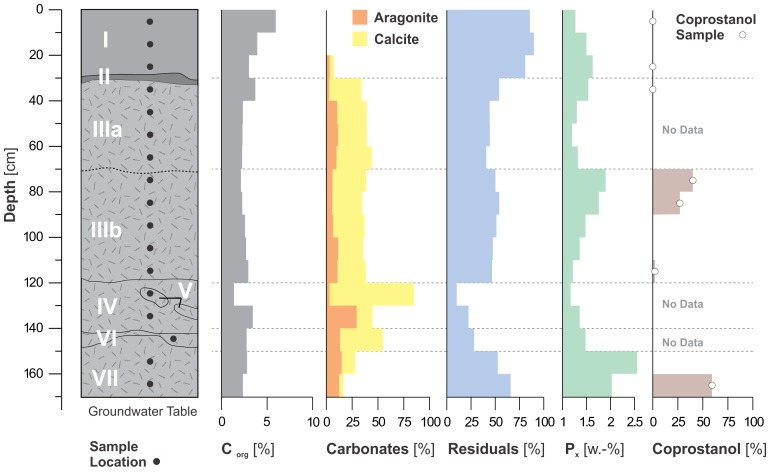
Stratigraphy, sampling sites and geochemical analyses of the profile at SM1. Residuals are mostly silt with small (<5%) amounts of clay and sand. Coprostanol is expressed as percentage of total detected steroids (see also Fig. 8).

Most of the SM1 site (Units III to VII) was deposited during the middle Holocene. There is considerable variation in the nature and taphonomic state of the material in these units. Shell fragments are the dominant material throughout, but in some levels concreted slabs occur with groundwater having prompted localized leaching, partial dissolution and re-precipitation of the calcium carbonate [47]. This is particularly noticeable in the upper portion of Unit III and in the lenses that constitute Units V and VI. Units IV and VII are largely unconsolidated and show less evidence of shell dissolution and recrystallization. The variable state of the shells is confirmed by both the geochemical results and the thin-section analysis, which show mixed burnt and unburnt shell fragments (Fig. 4a and 6b,c). It is likely that some of the recrystallization noted in the shell fragments is related to anthropic burning, with many of the animal bones also showing evidence of exposure to fire. The coupled analysis of thin sections and XRD shows that well-preserved fragmented shells are composed of aragonite, with many others having been completely transformed into micrite (microcrystalline calcite) (Fig. 6 and 7). Micritic shells have probably formed by exposure to heat as conversion of biogenic aragonite to calcite can occur at temperatures as low as 200°C [48], with a total transformation after 1 hour at 330°C [49]. XRD analysis also shows that about half of the material that constitutes the site is calcium carbonate which, in its majority, is derived from shells as there are no important sources of CaCO_3_ in the LM.

**Figure 8:**
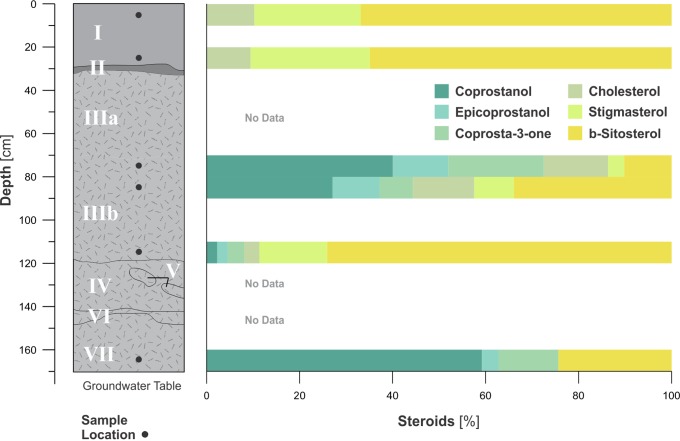
Steroid inventory of SM1. Single steroid abundance is expressed as percentage of total steroid amount. Cholesterol, ß-stigmasterol and b-Sitosterol occur in meat and plant tissues, while coprostanol, epicoprostanol and ß-coprostan mostly result from the digestion process of omnivores (for method details and IUPAC names see Text S1).

The analysis of steroids as specific markers for faeces points to omnivore sources, with coprostanol, a biomarker for human presence [50]–[53], dominating the main steroid fraction in the SM1 profile (Fig. 7, 8). Significant enrichment of coprostanol within the steroid pattern, preferentially produced by the human digestion apparatus, is found in zones III and VII. According to similar findings in Amazonian anthrosols [50], these data reveal an elevated input of human faeces in the shell midden. The shell midden also has a high content of benzene polycarboxylic acids (BPCAs), which serve as markers for black carbon, the residue of incomplete biomass combustion [45], [46]. The average black carbon content in Units III-VII is about 2.3 times greater than in the paleosol (Table 2) and its contribution to organic matter increases with depth. However, the content of black carbon relative to organic matter is higher in the paleosol than in the mound. Sediment multi-element analysis (Table 3) shows a sharp difference in the element assemblage between the paleosol and the midden due to the relative amounts of P, Ca, Al and Si; and between units I and units III–VII mainly due to the relative amounts of shell derived carbonates. The highest values of P are found at the bottom of the profile (unit VII), which coincides with the lowest values of Ca.

The faunal assemblage from SM1 is overwhelmingly dominated by both burnt and unburnt whole and fragmented apple snail shells (Fig. 4b). Ethnographic and archaeological data demonstrate human consumption of *Pomacea* snails and other relatively large gastropods in the region [54], [55]. In addition, a wide range of fragmented vertebrate faunal remains was also recovered from the shell midden (Units III–VII), including brocket deer (*Mazama* sp.) and marsh deer (*Blastocerus dichotomus*) bones (Fig. 4c,d) as well as bones of other mammals, fish, reptiles and birds – some of which were burnt (Table S3). Bones and shells are best preserved and most diverse in Unit VII, at a depth of 140–170 cm, associated with middle Holocene radiocarbon determinations. Two conjoining pieces of a marsh deer long-bone found at a depth of 160 cm show distinctive signs of an impact fracture (Fig. 4c). A potential item of material culture was found at 140 cm, a piece of fired clay upon which several parallel lines had been incised (Fig. 4e). Only two *Pomacea* opercula were recorded within the Unit III sample of several hundred shells. As is so often the case in archaeological faunal assemblages, some of the remains have probably been introduced by natural processes. The small numbers of Subulinidae microsnails and Streptaxidae macrosnails (*Streptartemon* sp.) found do not constitute human food waste; these snails probably lived and died within the boundary of the site. Additionally, very small reptile and fish bones may have been deposited by predators such as raptors – as noted by Langstroth [34].

Micromorphological analysis of the sediments confirms that the fragments of burnt earth (clay) found in varying quantities throughout the midden are largely of biogenic origin, with some wasps’ nests being clearly identifiable (Fig. 4a and Fig. 6d).

## Discussion

Our combined data for elevated concentrations of phosphorus, coprostanol and black carbon, along with the identified faunal assemblage and the stratigraphy at Isla del Tesoro and the consistency of the radiocarbon ages for Isla del Tesoro, SM2 and SM3, provide a strong case for the human origin of the three sites. Sediment multi-element analysis (Table 3) shows levels of P, Ca, Cu, Zn, Cl, S, Mn and Sr consistent with anthropogenic soils [56], with absolute P concentrations higher by a factor of 10 compared with anthropogenic plaggen soils [57] and within the same range as a midden composed of bones, shells and fish remains [58]. The negative correlation between P and Ca indicates that *Pomacea* is not the source of P, which probably comes from other anthropogenic inputs, as is often the case in archaeological sites [58].

The BC contribution to organic matter increases with depth and can be related to elevated inputs of burnt residues and/or to a preferential preservation of BC relative to other more labile organic matter constituents. However, the high concentration of total organic matter in the deeper part of the profile, indicating good preservation conditions, makes high depositional inputs of BC into zones III-VII a more likely explanation. Co-accumulation of BC together with P is a common feature in anthropogenic soils [59].

The absence of lithic artefacts in the region is to be expected due to the absence of stone resources in the surrounding landscape. Our data suggest that similar forest islands previously described as formed by predatory bird activity [34], could well be anthropogenic middens too. The putative occurrence of wildfires as the cause of fire damage is ruled out by the presence of discrete, concentrated burnt shell lenses in Units V and VI (Fig. 3, Fig. 4f). Birds are excluded as the main contributor to the formation of the middens based on the absence of feathers and eggshell fragments. Two species of South American birds prey on *Pomacea* snails as an important part of their diet: the Snail Kite (*Rostrhamus sociabilis*), which feeds exclusively on *Pomacea*, and the Limpkin (*Aramus guarauna*), which feeds mainly, but not exclusively, on *Pomacea* [60]. If the early shell mounds had been formed by non-anthropogenic processes, i.e. bird feeding habits as suggested by Langstroth [34], these are the species that could have been responsible for the shell accumulation. As these species are found nowadays in the LM [61], [62] similar sites should be forming at present in this or similar regions. However, no “modern” natural shell mounds have been reported in the LM or in other areas where Snail Kites or Limpkins are present. Whereas, the micro morphology and stratigraphy of Isla del Tesoro are quite similar to those of other anthropogenic shell middens elsewhere [63].

Although Units III to VII form a continuous midden with no interruptions of culturally-sterile layers, the occurrence of numerous detectable lenses and patches of localized burning suggests that the use of SM1 was intermittent, and possibly seasonal. Apple snails are most easily collected in the dry season when water levels are relatively low (Philip Darby, personal communication). Some *Pomacea* snails are found at the ground surface along the periphery of shallow ponds even when aestivating during the dry season [64], [65]. The prevalence of burnt wasps’ nests (Fig. 5d, 6d) may also indicate seasonal reoccupation. Wasps tend to build their nests during hiatuses in occupation, and their nests are eventually burned when the site is re-occupied.

Evidence from SM1 would suggest that the site was used as a food processing area where vertebrate and invertebrate prey was brought for preparation, cooking and consumption. The fact that only two opercula were found among a sample of several hundred shells suggests that *Pomacea* shells do not represent an *in situ* death assemblage. Apple snails are hardy molluscs that are able to survive through periods of extreme drought by sealing off the aperture with their operculum, burying into the mud and ‘aestivating’ for months or even years until the rains return [66], [67]. Thus, apple snails that entered aestivation but never re-emerged would be found with their opercula *in situ*, and even in heavily fragmented deposits numerous operculum fragments should be present. Given the scarcity of opercula also within unburnt assemblages, it could be possible that the snail meat was extracted from the shell and cooked or transported elsewhere. The higher value of BC and the even higher values of C_org_ in SM1 relative to the paleosol suggest that SM1 could have been a waste dumping site where fires were frequent. The shell midden formed roughly during the same time span as the paleosol, but it is about 10 times thicker than the paleosol. Therefore, the accumulation of BC and C_org_ per unit of time in the shell midden was, respectively, about 20 and 30 times higher than in the paleosol. This suggests that there were many more fire events and organic inputs in SM1 than in the surrounding area. As in the case of the middle Holocene tropical site of San Jacinto I in lowland Colombia where molluscs were abundantly consumed, the LM sites could have been occupied on a seasonal basis as logistical special-purpose locations for the procurement of temporarily abundant resources such as apple snails [68].The predominance of apple snails in SM1 points to a subsistence economy dependent on low-energy but readily available and highly predictable resources [69]. In coastal Brazil and along some of the most important river valleys where 10,180 to 9,710 cal BP *sambaquis* are found [70], the predictability of resources permitted the occupation of *sambaquis* for more than 8,000 years [6]. In SM1, the rich set of additional faunal resources, including large prey, such as marsh deer and brocket deer, as well as smaller aquatic and terrestrial prey, suggest a broad diet range. Carbon and Nitrogen isotopic data from humans exhumed from riverine *sambaquis* show that, despite the abundance of shellfish, early *sambaqui* dwellers relied mainly on a terrestrial diet based on brocket deer and small animals [6]. Given the similarity between the faunal assemblage at Isla del Tesoro and the faunal resources found in early Holocene Brazilian *sambaquis*, it cannot be ruled out that, in Isla del Tesoro, apple snails were a complementary resource.

Forest islands in the LM are well known among modern indigenous populations and ranchers, as during seasons characterized by severe and prolonged floods they become very important refugia for many animals, including cattle. As the midden slowly accreted and a mound began to develop, SM1 would have become an increasingly prominent feature above the seasonally-inundated grasslands, providing refuge for plants and animals, and a valuable hunting site for people. The cores analysed from the other shell middens, SM2 and SM3 (Fig. 1b), offer preliminary stratigraphic and chronological support that SM1 is not locally unique. The great modification caused to the ancient landscape by middle Holocene river activity [22] indicates that the environment in which the shell mounds formed was not necessarily similar to the modern one. However, the presence of *Pomacea* snails along with swamp deer and small fish, suggests that Isla del Tesoro and probably SM2 and SM3 too, were not riverine shell middens but a new category of wetland shell mounds. These sites represent a novel setting, with no relation to coastal cultures, for South American early Holocene shell middens.

**Figure 9:**
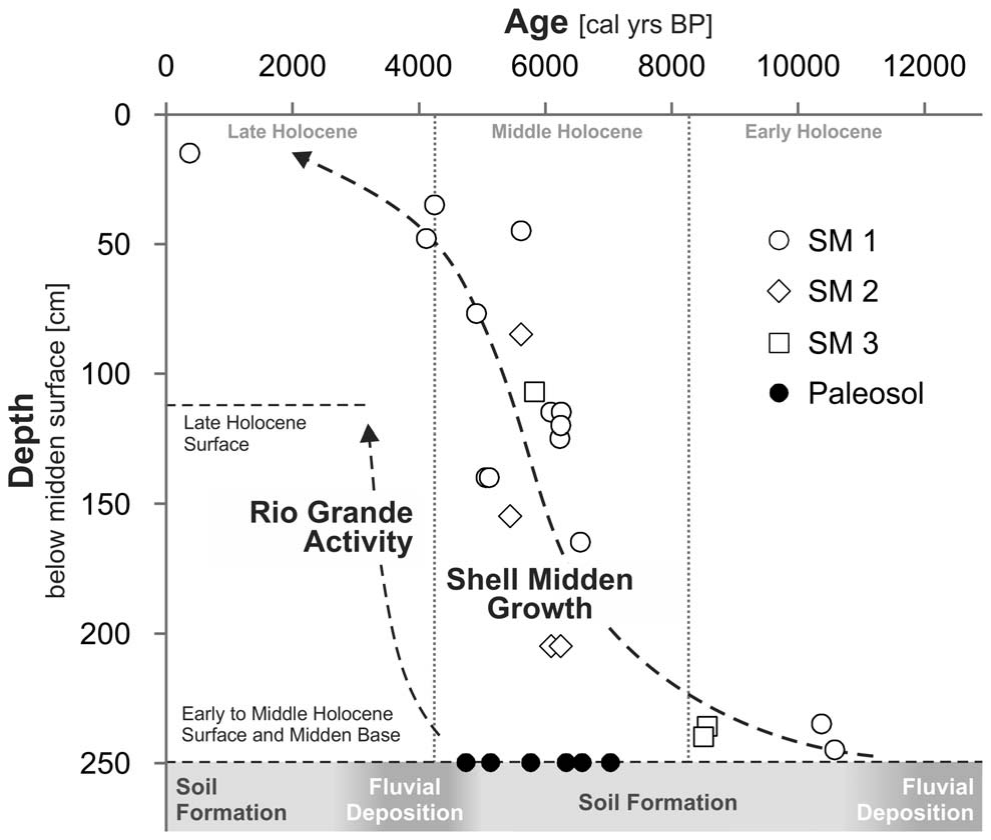
Relation between age and depth of the radiocarbon dated samples. Depth for the paleosol samples is in relation to the surface of SM1. Subdivision of the Holocene in early, mid and late is based on [78].

Therefore, rather than representing a late Holocene efflorescence of pre-Columbian agricultural communities in western Amazonia, as some have suggested [26], these LM forest islands demonstrate the emergent long-term trajectory of intensifying human utilization of this environment. The peopling of the LM probably followed the Pleistocene – Holocene transition and coincided with a period of soil formation. Paleo-ecological reconstructions suggest that this was a period characterized by a climate drier than today, with the landscape dominated by seasonally inundated savannahs [71]. Radiocarbon ages from the bottom of SM1 and SM3 (Fig. 9 and Table 1) indicate that humans settled in the LM during the early-Holocene. The shell mounds were used and accreted for approximately six thousand years. This process of prolonged low-level, seasonal occupation and deposition through time created a highly-localized feedback loop where anthropic deposition incrementally enhanced occupational space. The hiatus in the archaeological record after ∼4300 cal BP may indicate the sites were abandoned, possibly due to the climate shift towards wetter conditions that took place during the transition from mid- to late Holocene in tropical South America [71]–[74]. Our data, adding a new case to an already long list [68], [75], confirms that the middle Holocene was a time of great environmental changes in South America with important impacts on its population.

None of the shell middens studied was completely buried by sediments and it is likely that many other early Holocene forested archaeological sites exist in similar geomorphological contexts.

The presence of pottery and the faunal remains in the upper deposits (Unit I in Fig. 2), clearly points to a pre-Columbian re-occupation of SM1 by people belonging to the late-Holocene Earthmovers societies [25], [39]. The diverse range of fauna found in this topmost unit, including deer, tapir, armadillo, lungfish, swamp eel and apple snail (Table 2) reveals a varied diet incorporating large and small game as well as aquatic fauna, similar to material recovered from archaeological excavations at the late-Holocene sites of Loma Salvatierra and Loma Mendoza [55], [76], [77]. The elevation of the midden above the savannah created a preferential site for later re-occupation. Forest islands are known to have been important for both late pre-Columbian and ethno-historical societies of the LM [34], but what has not been recognized is the archaeological antiquity of at least some of these islands, and this casts a new light on the processes that lead to plant domestication, population growth, and the eventual emergence of complex societies in western Amazonia. The extensive networks of pre-Columbian monumental mounds, canals and causeways that the late Holocene societies built may represent the amplification of a pre-existing cultural landscape [26].

## Conclusions

This paper demonstrates, for the first time, early and middle Holocene human presence in western Amazonia. In the absence of preserved artefacts, geoarchaeological analysis can provide evidence of human presence. At Isla del Tesoro, this evidence includes black carbon, anthropically-accumulated faunal remains, and the chemistry of the sediments. The data from Isla del Tesoro, SM2 and SM3 and, in particular, their placement within a highly seasonal wetland landscape with scattered resources, suggest that early and middle Holocene groups here were very mobile. Such mobility will necessarily leave more ephemeral traces of human presence and consequently produce a very different archaeological record from sites in areas of high resource density. This visibility issue is compounded by later Holocene fluvial processes which have buried large expanses of the lowlands [22]. The noted absence of any documented Archaic sites in the Bolivian lowlands [12] may, therefore, be a consequence of poor visibility and the absence of adequate archaeological correlates for identifying hunter-gatherer open-air sites in a landscape completely devoid of lithic resources and other non-perishable materials.

It has become clear over the last two decades that late Pleistocene and early Holocene populations in South America successfully adapted to a wide range of environments. Yet, despite recognition of the diversity of environments where early human settlements have been documented, the western Amazonian lowlands continue to be considered challenging and unlikely to provide evidence of early habitation. The shell middens from the Llanos de Moxos provide just such evidence, prompting a reconsideration not only of the breadth of adaptive variation of early South American populations but also the manner in which sites are identified and interpreted.

## Supporting Information

Figure S1 **Chromatograms of masses 129 and 215 of a shell midden sample taken at 75 cm depth.** Soil matrix was dominated by shell debris. TLE is low at 0.06 mg g−1.
(TIF)

Figure S2 **Location of the paleosol samples reported in** **table 1** **and** **table 2** **.** Numbers indicate the code of the cores.
(TIF)

Table S1 **Trivial names and IUPASC names of analysed steroids and external standards used.**
(DOCX)

Table S2 **Total lipid extract (TLE) and steroid composition in shell midden SM1 and in soil samples from the surrounding savannah.**
(DOCX)

Table S3 **Faunal remains and burnt earth from SM1.**
(DOCX)

Text S1 **Methods of soil steroid and benzene-polycarboxylic acids detection.**
(DOCX)
