# Supplementary figures and images for: Early and Middle Holocene Hunter-Gatherer Occupations in Western Amazonia: The Hidden Shell Middens
Source: PLoS One. 2013 Aug 28;8(8):e72746. doi: 10.1371/journal.pone.0072746 (PMC3755986; doi:10.1371/journal.pone.0072746)

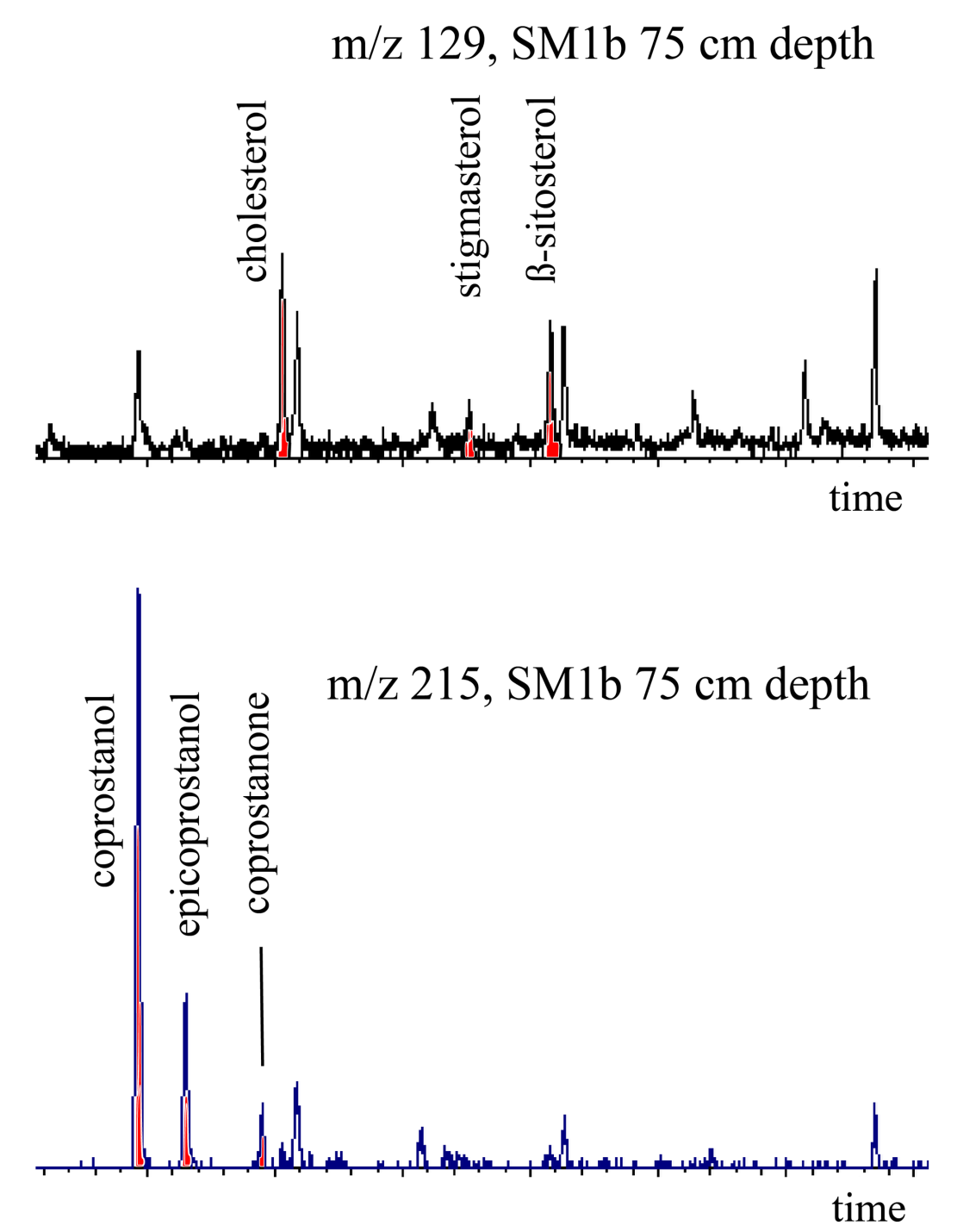

Supplement: Figure S1 — Chromatograms of masses 129 and 215 of a shell midden sample taken at 75 cm depth. Soil matrix was dominated by shell debris. TLE is low at 0.06 mg g−1. (TIF) [file pone.0072746.s001.tif]

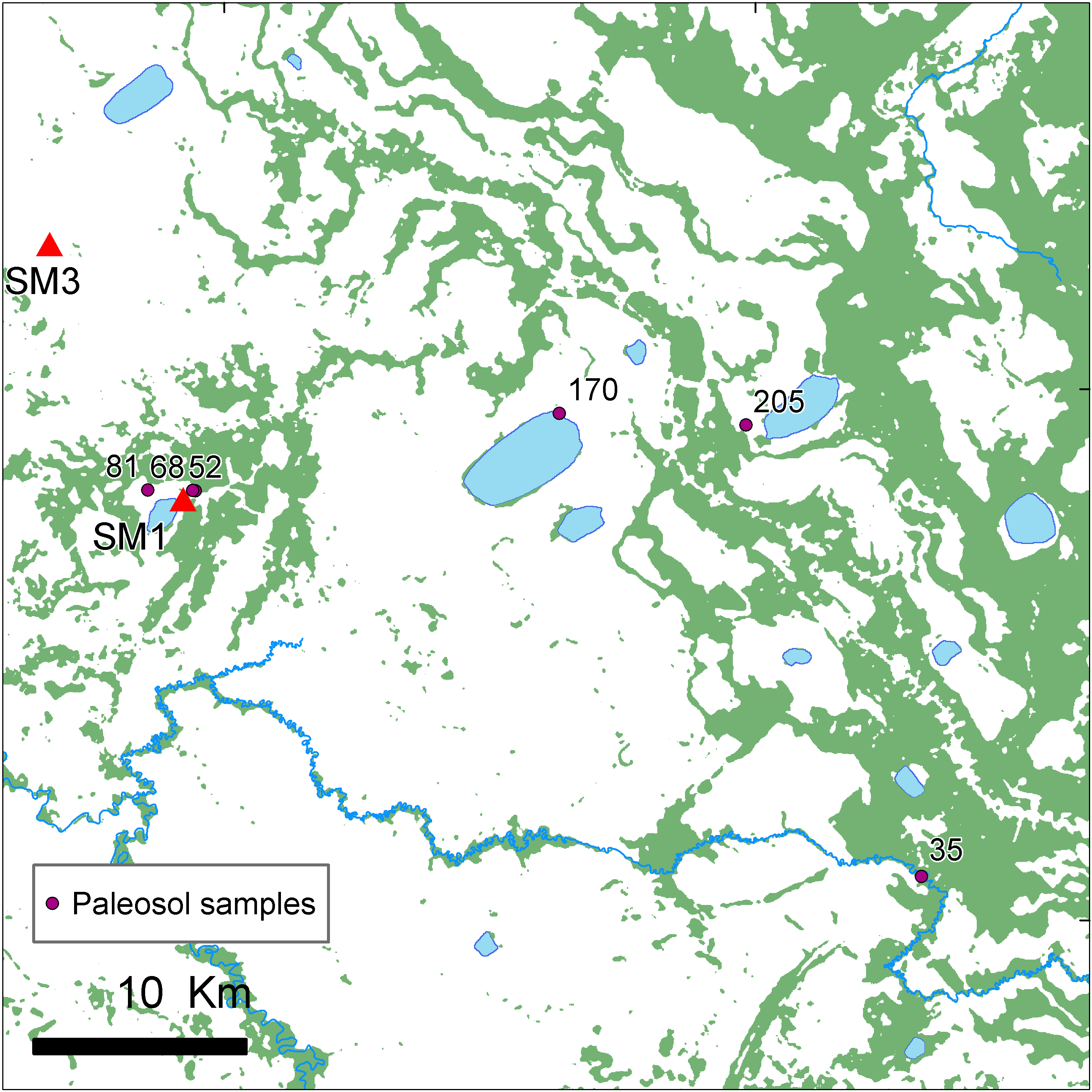

Supplement: Figure S2 — Location of the paleosol samples reported in table 1 and table 2 . Numbers indicate the code of the cores. (TIF) [file pone.0072746.s002.tif]
