# Supplementary material for: Early and Middle Holocene Hunter-Gatherer Occupations in Western Amazonia: The Hidden Shell Middens
Source: PLoS One. 2013 Aug 28;8(8):e72746. doi: 10.1371/journal.pone.0072746 (PMC3755986; doi:10.1371/journal.pone.0072746)
Supplement: Table S1 — Trivial names and IUPASC names of analysed steroids and external standards used. (DOCX) [file pone.0072746.s003.docx]

Table S1 Trivial names and IUPASC names of analysed steroids and external standards used. Ethylcoprostanol identified by elution series in environmental samples as reported by [6]

| **Steroids detected (trivial name)** | **IUPAC name (SciFinder.cas.org)** |
| --- | --- |
| Coprostanol | 5ß-cholestan-3ß-ol |
| Epicoprostanol | 5ß-cholestan-3α-ol |
| ß-coprostanone | 5ß-cholestan-3-one |
| Cholesterol | cholest-5-en-3ß-ol |
| Ethylcoprostanol * | 24ß-ethyl-5ß-cholestan-3ß-ol |
| Campestanol | 24ß-methyl-5α-cholestan-3ß-ol |
| ß-Stigmasterol | 24ß-ethylcholest-5,22-dien-3ß-ol |
| ß-Sitosterol | 24ß-ethylcholest-5-en-3ß-ol |
|  |  |
| **External standard (trivial name)** | **IUPAC name (SciFinder.cas.org)** |
| Coprostanol | 5ß-cholestan-3ß-ol |
| Epicoprostanol | 5ß-cholestan-3α-ol |
| α-coprostanone | 5α-cholestan-3-one |
| Cholesterol | cholest-5-en-3ß-ol |
| campesterol | 24α-methyl-5-cholesten-3β-ol |
| α-stigmastanol * | 24α-ethyl-5α-cholestan-3β-ol |
| α-cholestan | 5α-cholestane |
| dihydrocholesterol | 5α-cholestan-3ß-ol |
| hydroxycholesterol | 5-cholestene-3β,20α-diol |

* Substances not reliably separated
