# Supplementary material for: Early and Middle Holocene Hunter-Gatherer Occupations in Western Amazonia: The Hidden Shell Middens
Source: PLoS One. 2013 Aug 28;8(8):e72746. doi: 10.1371/journal.pone.0072746 (PMC3755986; doi:10.1371/journal.pone.0072746)
Supplement: Table S2 — Total lipid extract (TLE) and steroid composition in shell midden SM1 and in soil samples from the surrounding savannah. (DOCX) [file pone.0072746.s004.docx]

Table S2 Total lipid extract (TLE) and steroid composition in shell midden SM1 and in soil samples from the surrounding savannah. Soils were sampled at 15 to 150 m distances from SM1 in westerly and easterly directions. Steroids analysed via GC-MS, given as a percentage of the sum of steroids identified in each sample (nd = not detected; empty lines = samples not analysed).

| **Depth** | **Site** | **TLE** | **Coprostanol** | **Epicoprostanol** | **ß-Coprostanone** | **Cholesterol** | **Ethylcoprostanol** | **Campestanol** | **ß-Stigmasterol** | **ß-Sitosterol** | |
| --- | --- | --- | --- | --- | --- | --- | --- | --- | --- | --- | --- |
| [cm] |  | [mg g-1] | % of total sterol peak intensity | | | | | | | |  |
| 5 | SM1 | 0.78 | 0.03 | nd | nd | 10.3 | nd | nd | 22.8 | 66.9 | |
| 25 | SM1 | 0.34 | Nd | nd | nd | 9.5 | nd | nd | 25.6 | 64.9 | |
| 75 | SM1 | 0.06 | 40.1 | 11.9 | 20.5 | 13.9 | nd | nd | 3.4 | 10.2 | |
| 85 | SM1 | 0.06 | 27.1 | 10.1 | 7.2 | 13.2 | nd | nd | 8.5 | 33.9 | |
| 115 | SM1 | 0.06 | 2.2 | 2.4 | 3.6 | 3.2 | nd | nd | 14.6 | 74.1 | |
| 165 | SM1 | 0.09 | 59.2 | 3.6 | 12.9 | nd | nd | nd | 0.0 | 24.3 | |
|  |  |  |  |  |  |  |  |  |  |  | |
| 15 | savannah | 0.95 | Nd | nd | nd | nd | nd | nd | 50.7 | 49.3 | |
| 35 | savannah | 0.21 | Nd | nd | nd | 4.6 | nd | 35.6 | 22.0 | 37.7 | |
| 95 | savannah | 0.11 | Nd | nd | nd | 7.5 | nd | 19.9 | 26.4 | 46.3 | |
| 15 | savannah | 1.76 | Nd | nd | nd | nd | nd | nd | 46.9 | 53.1 | |
| 95 | savannah | 0.39 | Nd | nd | nd | 6.8 | nd | 25.5 | 27.3 | 40.4 | |
| 35 | savannah | 0.27 | nd | nd | nd | 7.5 | nd | nd | 30.7 | 61.8 | |
| 85 | savannah | 0.20 | nd | nd | nd | 11.8 | nd | nd | 17.8 | 70.4 | |
| 35 | savannah | 0.38 | nd | nd | nd | nd | nd | 35.2 | 28.2 | 36.7 | |
| 95 | savannah | 0.12 | nd | nd | nd | nd | nd | nd | 30.6 | 69.4 | |
