# Supplementary material for: Early and Middle Holocene Hunter-Gatherer Occupations in Western Amazonia: The Hidden Shell Middens
Source: PLoS One. 2013 Aug 28;8(8):e72746. doi: 10.1371/journal.pone.0072746 (PMC3755986; doi:10.1371/journal.pone.0072746)
Supplement: Table S3 — Faunal remains and burnt earth from SM1. (DOCX) [file pone.0072746.s005.docx]

**Table S3** **Faunal remains and burnt earth from SM1.**

| **Depth below surface (cm)** | **Unprocessed sample weight (g)** | **Burnt earth weight (g)** | ***Pomacea* spp*.* shell fragments (NISP)** | ***Pomacea* spp*.* shell weight (g)** | **Landsnail (all complete)** | **Vertebrate fauna** | **Material culture** |
| --- | --- | --- | --- | --- | --- | --- | --- |
| 0-10 | 221 |  | 5 | <1 |  |  |  |
| 10-20 | 424 | 194 | 4 | <1 |  | Marsh deer (*Blastocerus dichotomus*) longbones. Caiman (*Caiman yacare*) skull fragments. Unidentified reptile. Fish vertebrae (n=2). Fragments of bone <5mm (n=30) |  |
| 20-30 | 465 | 167.5 | 8 | 1.5 |  | Brocket deer (Mazamasp.) metatarsal. Tapir (*Tapirus terrestris*) incisor. Armadillo (*Euphractus* sp.) carapace scales. Unident. mouse tibia. Caiman (*Caiman yacare*) skull fragments. Unident. reptile longbones. Lungfish (*Lepidosiren paradoxus*) dentaries. Swamp eel (*Synbranchus* sp.) dentaries. Unident. bird vertebra. Unident. fish vertebrae.Fragments of bone <5mm (n=51); including 2 calcined and 5 blackened bones (indicating burning) | Earthenware potsherds (n=2) (Fig S2F). 2 bone tools (Fig S2E) |
| 30-40 | 282 | 4.5 | 66 | 5 | 1 Subulinidae | Small bones/bone fragments (n=38), including 1 fish vertebra as well as some small longbone shaft fragments indicating non-fish fauna; 3 bones are burnt glossy black |  |
| 40-50 | 1064 | 31.5 | cemented clasts | 512.5 | 3 Subulinidae | Bird vertebra (n=1). Unident. bone (n=4); 1 burnt |  |
| 50-60 | 377 | 5 | cemented clasts | 272 |  | Unident. bone fragments (n=2) |  |
| 60-70 | 354 | 0.5 | cemented clasts | 251 |  | Brocket deer (*Mazama* sp.) mandible fragment (Fig 3F) |  |
| 70-80 | 683 |  | cemented clasts | 226.5 |  |  |  |
| 75-85 | 295 |  | cemented clasts | 251 |  |  |  |
| 80-90 | 355 | 13.5 | cemented clasts | 169.5 |  |  |  |
| 80-100 | 338 | 4 | cemented clasts | 117 |  |  |  |
| 100-110 | 393 | 2.5 | cemented clasts and loose fragments | 89 |  | Unident. bone fragments (n=2) |  |
| 110-120 | 400 | 2.5 | cemented clasts and loose fragments | 109.5 | 1 Subulinidae | Unident. reptile vertebra (n=1). Unident. bone (n=3) 1 burnt |  |
| 120-130 | 905 |  | cemented clasts | 889 |  |  |  |
| 130-140 | 781 |  | cemented clasts and loose fragments; some burnt | 580.5 | 2 Streptaxidae | Unident. bone fragment (n=1) |  |
| 140-150 | 648 |  | cemented clasts and loose fragments; some burnt | 520.5 |  | Unident. fish vertebrae; 4 burnt | Angular burnt earth with incised parallel lines |
| 150-160 | 302 |  | loose fragments; some burnt | 77.5 |  | Marsh deer (*Blastocerus dichotomus*) long-bones. Unident. fish vertebrae, spines and teeth (n=43) |  |
| 160-170 | 143 |  | loose fragments; some burnt | 41 |  | Unident. fish vertebrae and spines (n=10). Unident. <5mm bone fragments (n=49); 15 burnt |  |
