# Supplementary material for: Early and Middle Holocene Hunter-Gatherer Occupations in Western Amazonia: The Hidden Shell Middens
Source: PLoS One. 2013 Aug 28;8(8):e72746. doi: 10.1371/journal.pone.0072746 (PMC3755986; doi:10.1371/journal.pone.0072746)
Supplement: Text S1 — Methods of soil steroid and benzene-polycarboxylic acids detection. (DOCX) [file pone.0072746.s006.docx]

**Supporting information**

**Analytical Methods:**

***Soil steroid analysis***

**Aim**

The purpose of this analysis is to determine whether the geochemical analyses of steroids in shell midden SM1 provides additional support to the hypothesis of early Holocene human presence at the site. Among the steroids, 5ß-stanols in fresh water [1], lake sediments [2,3] and soil [4-6] were found to be good indicators of past human presence. Several studies have also shown a contribution from domesticated animals [7,8] and microbial degradation in soil [5] to the 5ß-steroid signal. Coprostanol (5ß-cholestan-3ß-ol), epicoprostanol (5ß-cholestan-3α-ol) and coprostanone (5ß-cholestan-3-one) dominate when a significant input of human or domesticated animals faeces occurs [1,3,5,6]. Their significance can be evaluated by comparing faeces derived steroids to their precursors, e.g. cholesterol and phytosterols [1,5]. Therefore, we have analysed these markers as additional evidence for a contribution of human or livestock faeces to the organic matter in the shell midden.

**Methods**

The method used is along the lines of that described in [1,4]. A detailed description of how the analysis was performed is outlined here. We chose a solvent mixture with a slightly lower polarity for the extraction of the steroids in order to avoid the extraction of compounds that would have to be excluded afterwards. Hence, the total lipid extract (TLE) could be analysed for its steroid content (after derivatization) without purification. This extraction solvent mixture has proved efficient in analysing various lipid compounds [9].

Lipid extraction was done by accelerated solvent extraction (ASE350, DIONEX, Idstein, Germany) with dichloromethane: methanol (9:1, v/v). The total lipid extract (TLE) was derivatized with BSTFA employing pyridine as catalyst (at 70°C for 3 hours) for steroid analysis. Steroid patterns were then analysed by gas chromatography – mass spectrometry (Agilent 5971 GC-MSD; HP5-MS column, 30 m length, 0.25 mm inner diameter, 0.25 µm film; temperature program from 70° C to 320 °C with 7° C per minute). As the gas chromatography already yielded baseline separated steroid signals (e.g., Fig. S1), we refrained from a fractionation of the TLE. Steroids were identified by typical mass spectra (lipidlibrary.aocs.org), elution series and comparison to literature data [6]. We found coprostanol, epicoprostanol, ß-coprostanone, cholesterol, ethylcoprostanol, campestanol, stigmasterol and ß-sitosterol in detectable amounts (e.g., Fig. S1). Quantity and quality was checked by external standards employing α-cholestan, coprostanol, epicoprostanol, cholesterol, dihydrocholesterol (=5α-cholestan-3ß-ol), α-coprostanone, campesterol, stigmasterol, ß-sitosterol, α-stigmastanol (=α-ethylcoprostanol, coeluting with ß-ethylcoprostanol), hydroxycholesterol (IUPAC names provided in Table S1). Precision was on average 13 %. Response of standard materials, referring to α-cholestan, varied between 0.88 for dihydrocholesterol to 2 for coprostanol. The latter implies that there is an uncertainty of a factor of 2 of the total peak area of coprostanol, i.e., its contents may be twice as high in the sample as conservatively indicated by standard analyses. However, in this first test, for verifying the occurrence of residues it was considered sufficient if the peaks found were above the detection limit. Due to the unsolved matrix effects at the time of the analysis, we refrained from a quantification of each steroid and expressed single steroid contents (peak areas) as a percentage of the sum of the detectable steroids (summed peak areas).

**Results & Discussion**

From shell midden SM1 and its surroundings a total of fifteen samples have been analysed: two samples from Unit I, three from Unit III, one from unit VII and nine samples from the surrounding savannah (Table S2). Savannah samples were taken to be used as an external reference. Although the amounts of total lipid extracts (range 0.06 to 1.76 mg g^-1^) were at least 10 times lower than is usual in recent topsoils [10], we found a well detectable signal of coprostanol in SM1 below -75 cm (units III and VII). The ratio of human-related coprostanol to plant-derived steroids was clearly elevated in the shell midden samples and comparable to observations made in anthropogenically influenced environments [1,5].

Bull et al. [4,11] suggested that the ratio between coprostanol and its 5α isomer is the most reliable ratio in order to detect human faeces. As the 5α isomer originates from microbial degradation (=natural background) this ratio makes it possible to detect if amounts of coprostanol are significantly higher than in the natural background. However, given the absence of 5α isomer in our sample, we could not calculate this index.

The total absence of coprostanol in the savannah reference samples and in unit I, together with the abundance of phytosterols and one phytostanol (Table S2), suggests that it is very unlikely that the input of human-related stanols in the shell midden has any other source than omnivorous/human faeces.

***Detection of benzene-polycarboxylic acids as markers for black carbon***

The oxidation of black carbon (BC) to benzene polycarboxylic acids (BPCA) was carried out following Brodowski et al. [12]. Samples (ca. 5 mg carbon) were hydrolyzed with trifluoroacetic acid for metal elimination (105 °C, 4 h). The residue was oxidized with 65% HNO_3_ (170 °C, 8 h), BPCAs were subsequently purified using a cation exchange column (Dowex 50 W X 8, 200–400 mesh, Fluka, Steinheim, Germany). The individual BPCAs were then converted to trimethylsilyl derivatives, separated by gas-chromatography using an Equity-5 column (30 m x 0.25 mm i.d., 0.25 µm film thickness; Supelco, Steinheim, Germany), and detected via flame ionization (Agilent 6890 gas-chromatograph). Citric acid was used as a first internal standard for BPCA quantification and added immediately before the cation exchange step. Biphenylene-dicarboxylic acid was used as a second internal standard to quantify the recovery of citric acid (recovery 70–95%). The BPCA yields were corrected for CO_2_ loss and insufficient conversion of BC to BPCAs by a factor of 2.27 [13], representing a conservative minimum of BC estimation [12].

References

1. Grimalt JO, Fernandez P, Bayona JM, Albaiges J (1990) Assessment of fecal sterols and ketones as indicators of urban sewage inputs to coastal waters. Environ Sci Technol 24: 357-363.

2. Meyers PA (2003) Applications of organic geochemistry to paleolimnological reconstructions: a summary of examples from the Laurentian Great Lakes. Org Geochem 34: 261-289.

3. D’Anjou RM, Bradley RS, Balascio NL, Finkelstein DB (2012) Climate impacts on human settlement and agricultural activities in northern Norway revealed through sediment biogeochemistry. Proc Natl Acad Sci U S A.

4. Bull ID, Evershed RP, Betancourt PP (2001) An organic geochemical investigation of the practice of manuring at a Minoan site on Pseira Island, Crete. Geoarchaeology 16: 223-242.

5. Birk JJ, Teixeira WG, Neves EG, Glaser B (2011) Faeces deposition on Amazonian Anthrosols as assessed from 5β-stanols. J Archaeol Sci 38: 1209-1220.

6. Shillito L-M, Bull ID, Matthews W, Almond MJ, Williams JM, et al. (2011) Biomolecular and micromorphological analysis of suspected faecal deposits at Neolithic Çatalhöyük, Turkey. J Archaeol Sci 38: 1869-1877.

7. Leeming R, Ball A, Ashbolt N, Nichols P (1996) Using faecal sterols from humans and animals to distinguish faecal pollution in receiving waters. Water Res 30: 2893-2900.

8. Gill FL, Dewhurst RJ, Dungait JAJ, Evershed RP, Ives L, et al. (2012) Archaeol – a biomarker for foregut fermentation in modern and ancient herbivorous mammals? Org Geochem 41: 467-472.

9. Wiesenberg GLB, Schmidt MWI, Schwark L (2008) Plant and soil lipid modifications under elevated atmospheric CO2 conditions: I. Lipid distribution patterns. Org Geochem 39: 91-102.

10. Stevenson FJ (1994) Humus chemistry: genesis, composition, reactions. New York: Wiley. 496 p.

11. Bull ID, Lockheart MJ, Elhmmali MM, Roberts DJ, Evershed RP (2002) The origin of faeces by means of biomarker detection. Environ Int 27: 647-654.

12. Brodowski S, Rodionov A, Haumaier L, Glaser B, Amelung W (2005) Revised black carbon assessment using benzene polycarboxylic acids. Org Geochem 36: 1299-1310.

13. Glaser B, Haumaier L, Guggenberger G, Zech W (1998) Black carbon in soils: the use of benzenecarboxylic acids as specific markers. Org Geochem 29: 811-819.
